# Supplementary material for: A guide, cornerstone, and appetizer: An elicited metaphor analysis of Chinese university students’ perceptions of English language textbooks
Source: PLoS One. 2025 Mar 5;20(3):e0315292. doi: 10.1371/journal.pone.0315292 (PMC11882096; doi:10.1371/journal.pone.0315292)
Supplement: Attachment — Submitted filename: Response to Reviewers.docx [file pone.0315292.s001.docx]

Dear editor and reviewers,

We sincerely thank you and reviewers for the valuable comments, which have greatly helped us improve our manuscript. We have extensively revised the manuscript based on the feedback received. All changes are marked by Track Changes and highlighted for easier reading. Below are our responses to comments:

Comments from the editor:

1. Additionally, we kindly ask you to consider the following PLOS ONE criteria regarding qualitative and mixed-methods studies for publication. Your study should include the following information: "description of the sampling strategy, including rationale for the recruitment method, participant inclusion/exclusion criteria and the number of

participants recruited". Please check whether you have taken all those information into account or add any missing information.

Our response: Thank you for reminding us of this issue. We have double checked our manuscript and we hereby confirm that these aspects have been reported in section 3.1 (highlighted in the revised manuscript).

2. Thank you for stating in your Funding Statement: This study is partially supported by Faculty Reserve Grant (P0048130), Faculty of Humanities, The Hong Kong Polytechnic

University, Hong Kong SAR, China.

Please provide an amended statement that declares *all* the funding or sources of support (whether external or internal to your organization) received during this study, as detailed online in our guide for authors at http://journals.plos.org/plosone/s/submit-now.

Please also include the statement “There was no additional external funding received for this study.” in your updated Funding Statement. Please include your amended Funding Statement within your cover letter. We will change the online submission form on your behalf.

Our response: We have included the following amended statement within our new cover letter.

Updated statement: This study is supported by a Faculty Reserve Grant (P0048130), Faculty of Humanities at The Hong Kong Polytechnic University, a Start-up Fund for RAPs under the Strategic Hiring Scheme (P0048980) at The Hong Kong Polytechnic University, and a Pujiang Talent Program (21PJC014) awarded to the first author. There was no additional external funding received for this study.

3. Please include your full ethics statement in the ‘Methods’ section of your manuscript file. In your statement, please include the full name of the IRB or ethics committee who approved or waived your study, as well as whether or not you obtained informed written or verbal consent. If consent was waived for your study, please include this information in your statement as well.

Our response: We have updated this information in section 3.1 (see pages 10-11).

4. Please include captions for your Supporting Information files at the end of your manuscript, and update any in-text citations to match accordingly. Please see our Supporting Information guidelines for more information: <http://journals.plos.org/plosone/s/supportinginformation>.

Our response: We have included the captions for the two supporting information files at the end of the updated manuscript.

5. Please review your reference list to ensure that it is complete and correct. If you have cited papers that have been retracted, please include the rationale for doing so in the manuscript text, or remove these references and replace them with relevant current references. Any changes to the reference list should be mentioned in the rebuttal letter that accompanies your revised manuscript. If you need to cite a retracted article, indicate the article’s retracted status in the References list and also include a citation and full reference for the retraction notice.

Our response: Thank you for the reminder. We have checked the reference list to make sure it is complete and correct. None of the cited papers have been retracted.

Comments from reviewer 1:

(1) On page 35 the researchers describe their study as a “mixed-method investigation,”

but I am unclear on what part of the research design is quantitative. It might be that the researchers are referring to the quantitative analysis that they employed. In this case, my recommendation would be to specifically identify their analysis as mixed-methods.

Our response: Thank you very much for pointing out this problem. We have revised the data analysis part in the method (3.5) to explicitly demonstrate that our analysis employs mixed methods. Please see the revised sentences on page 16.

(2) On page 11 the researchers mention excluding responses that did not contain metaphors. I thought it would have been helpful to provide an example.

Our response: Thank you for this suggestion! We have provided an example following this statement. Please see page 11.

(3) On page 9 the researchers write, “The study suggests modifications and additional materials to better cater to the needs of EFL graduate students.” I am not sure whether “the study” being referred to is the current study or Shi’s (2022) study.

Our response: Thank you for pointing out this problem. We have clarified it by adding Shi (2022) into the sentence. Please see page 8.

(4) When looking at Table 3 on its own, I did not know what the red and green highlights

meant. While this is explained in the body of the document, a note could be added to the table for the purposes of clarification.

Our response: Thank you for this concrete suggestion. We have added notes for Table 3. Please see page 26.

(5) In the reference section, there are some minor APA Style formatting errors.

The most consistent one was not including DOI links, which would be

easy to resolve.

Our response: Thank you for pointing out this problem. We have changed the format to Vancouver style which is required by the journal. We have also added DOI links to references for all those that have a DOI.

(6) One grammatical issue I noted was the occasional use of commas when not needed (see selected examples below).

Unnecessary comma examples

Page 6 - “when we lose our way in English learning, textbooks guide

us in the right direction”, textbooks are metaphorically portrayed as

guides

Page 7 – and evaluations of textbook content (Deignan, 2017;

Deignan & Semino, 2019), to name a few.

Page 17 - Learners may assimilate knowledge at a standard pace

(walking) (refer to case 1 in Extract 1), or strive to keep pace with

others (running).

Our response: Thank you for listing these examples in details. We have addressed these problems one by one. Please see pages 6, 7 and 18.

(7) Another grammatical issue I noted was the occasional awkward

wording, which caused me to experience issues with comprehension

(see selected examples below).

Awkward wording examples

Abstract - The synergy of discourse analysis and statistical analysis

suggests learners’ physical and emotional engagement with English

language textbooks.

Page 12 - a few responses which can hardly be categorized from any

dimension

Page 8 - learner disaffection spans four categories

Page 16 - total of 75 metaphors constitute to these Conceptual

Metaphors

Our response: Thank you for listing these examples in details. We have rephrased these sentences one by one. Please see pages 3, 11 and 17.

Comments from reviewer 2:

(1) The study employs a sound methodology using Conceptual Metaphor Theory (CMT) to analyze Chinese EFL students' metaphors for English textbooks. However, while the data is intriguing, the manuscript lacks deeper theoretical engagement with recent metaphor research. Updating and expanding the literature review to include more recent works would strengthen the paper's relevance.

Our response: Thank you for this suggestion. We have updated and expanded the literature review with more recent works to address this issue. Please see our changes highlighted in introduction and theoretical background (pages 4 to 10)

(2) The elicited metaphor analysis (EMA) is well executed, but the coding process for metaphors and emotional valence needs more detail.

Our response: Thank you very much for these concrete suggestions! We have added examples to illustrate the coding process of metaphors. Please see the updated sections 3.3 on pages 12-14. We have elaborated the coding process for emotional valence and added an example. Please see the updated 3.4 on pages 14-16.

(3) While Krippendorff's alpha scores indicate reliability, additional transparency on the disagreements during coding would be beneficial.

Our response: Thank you very much for this suggestion. We have added disagreed examples to improve the transparency. Please see the updated sections 3.3 and 3.4 on pages 14-16.

(4) The study makes an original contribution by expanding metaphor analysis to domains/frames rather than focusing solely on mental spaces. However, the conclusions could benefit from further linking the findings to pedagogical applications or practical suggestions for improving textbook design and use.

Our response: Thank you for this suggestion. We have elaborated our pedagogical applications in the conclusion. Please see our revised section on pages 34-35.

(5) The statistical analysis (Chi-Square Test of Independence) is appropriate for examining the relationship between metaphor sources and emotional valence.

However, a discussion of the effect size could provide better insight

into the practical significance of the findings.

Our response: Thank you for this comment! We have integrated the discussion of effect size into discussing practical implications. Please see our revised section 5.3 on pages 31-32.

(6) The metaphorical insights are well connected to the learners' attitudes, but the pedagogical implications need further exploration. While the discussion mentions implications for EFL learning and textbook design, it would benefit from a more robust exploration of how these metaphorical insights could be applied by educators.

Our response: Thank you for this comment! We have elaborated the discussion of practical implications for educators and language learners. Please see our revised section 5.3 on pages 31-33.

(7) The ethics statement is clear, and the informed consent process is

appropriate.

Many thanks for this comment!

(8) In conclusion, this manuscript offers a promising exploration of metaphorical perceptions in EFL learners, but could benefit from further engagement with recent research and more in-depth discussion of the practical applications of the findings.

Our response: Thank you for your concrete suggestions. We have included more recent research and elaborated discussion of the practical applications.

We found that these insightful suggestions significantly enhance our manuscript. We hope our improved version could meet the requirements of this journal. Thank you again for your valuable time in reviewing our manuscript!

Best wishes,

Authors
